# Supplementary material for: ALUMINUM RESISTANCE TRANSCRIPTION FACTOR 1 (ART1) contributes to natural variation in aluminum resistance in diverse genetic backgrounds of rice (O. sativa)
Source: Plant Direct. 2017 Oct 16;1(4):e00014. doi: 10.1002/pld3.14 (PMC6508803; doi:10.1002/pld3.14)
Supplement: Supplementary file 1 [file PLD3-1-e00014-s001.docx]

**Supplemental Figure S1: Fine-mapping of the Al resistance QTL *Alt12.1*.** Individual rice lines were clustered into genotypic groups according to their recombination breakpoints. The ‘informative marker’ column indicates the markers used to define recombination breakpoints. The ‘genotypic class’ column shows the nomenclature of each recombinant haplotype group; ‘RRG mean’ shows Al resistance phenotypic performance defined as the mean relative root growth (RRG) for each genotypic class. The ‘number of families’ column indicates the number of F_3_ lines that contributed F_4_ offspring in each genotypic class. The ‘N’ column shows the total number of F_4_ plants phenotyped in each genotypic class. The ‘T-K HSD’ column indicates genotypic classes showing significant phenotypic differences based on Tukey’s pairwise comparisons (*p* < 0.05).


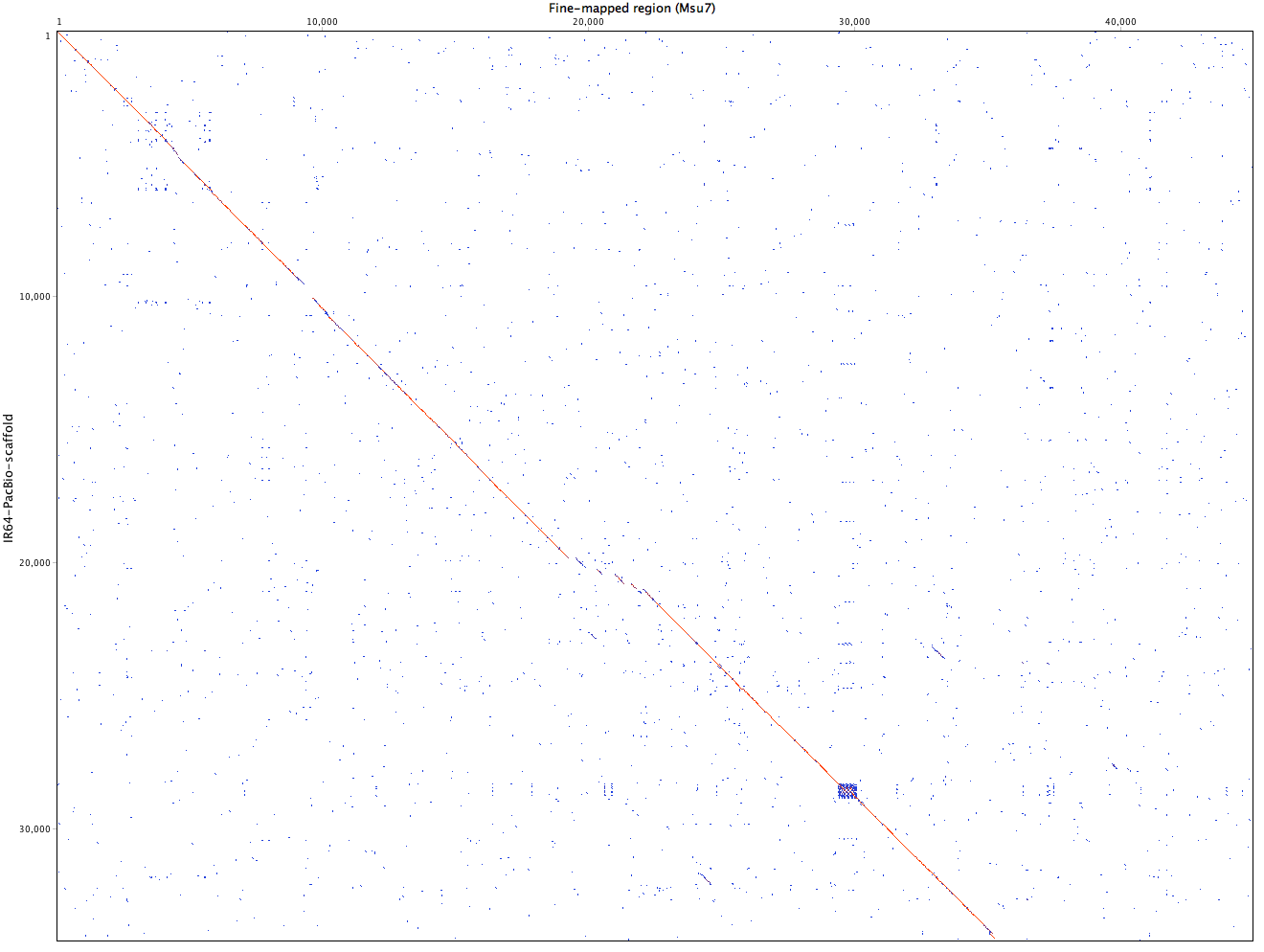


**Supplemental Figure S2: Alignment of the QTL *Alt12.1* fine-mapped region (Nipponbare reference genome) to IR64 (PacBio assembly).** The dot-plot shows a graphical representation of the similarity between two sequences. The long diagonal line indicates regions where the two sequences have substantial similarity. Breaks in the diagonal line indicates insertions or deletions. Parallel diagonal lines indicate repeats. Sequences were aligned using MAFFT (<http://mafft.cbrc.jp/alignment/software)>. Dot-plot was built using the EMBOSS tool *dottup* (<http://emboss.sourceforge.net/apps/release/6.5/emboss/apps/dottup.html)>.

**Supplemental Figure S3: Principal component analysis (PCA) of gene expression profiles**. (**A)** PCA on normalized RNA-Seq counts of Azucena and AZU_[IR6412.1]_ samples under control (0 μM Al^3+^ activity) and Al stress conditions (80 μM Al^3+^ activity) for 4 hours. Azu.CK: Azucena control samples (dark blue squares); AZU_[IR64]_.CK: NIL AZU_[IR6412.1]_ control samples (light red diamonds). Azu.Al: Azucena stress samples (dark blue circles); AZU_[IR64]_. Al: NIL AZU_[IR6412.1]_ stress samples (light red triangles). (**B**) PCA on normalized RNA-Seq counts of IR64 and IR64_[AZU12.1]_ samples under control (0 μM Al^3+^ activity) and Al stress conditions (80 μM Al^3+^ activity) for 4 hours. IR64.CK: Azucena control samples (dark red squares); IR64_[AZU]_.CK: NIL IR64_[AZU12.1]_ control samples (light blue diamonds). IR64.Al: IR64 stress samples (dark red circles); IR64_[AZU]_. Al: NIL IR64_[AZU12.1]_ stress samples (light blue triangles).


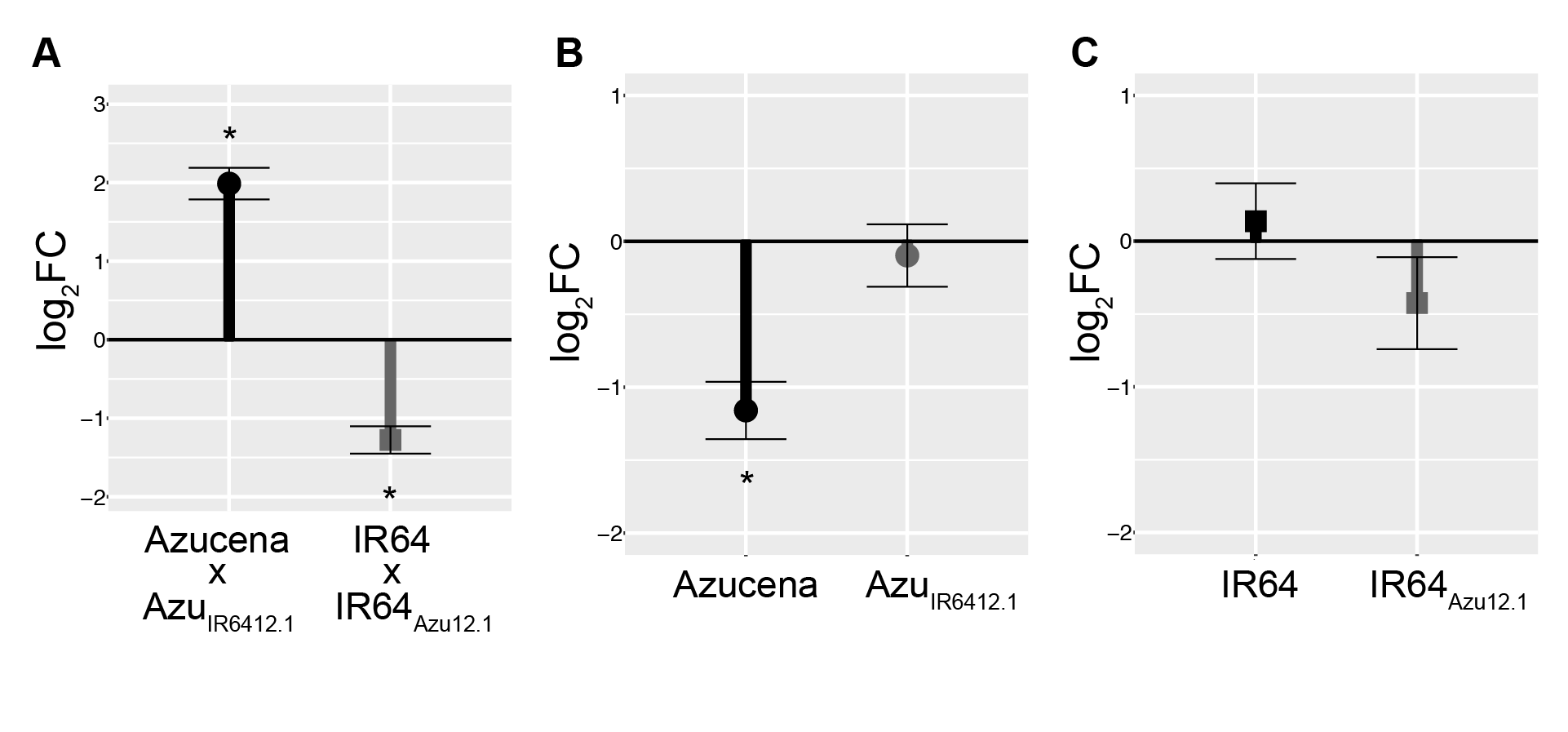
**Supplemental Figure S4: Differential expression of LOC_Os12g07340, located within the *Alt12.1* fine-mapped region, based on RNA-seq. (A)** LOC_Os12g07340 expression in each NIL *versus* its recurrent parent under control conditions (log_2_Fold-Change). **(B)** and **(C)**: RNAseq-based expression pattern for LOC**_**Os12g07340 in response to Al (log_2_Fold-Change) in each genotype, in **(B)** Azucena and **(C)** IR64 genetic backgrounds. An asterisk indicates statistically significant differential expression (*p* < 0.05).


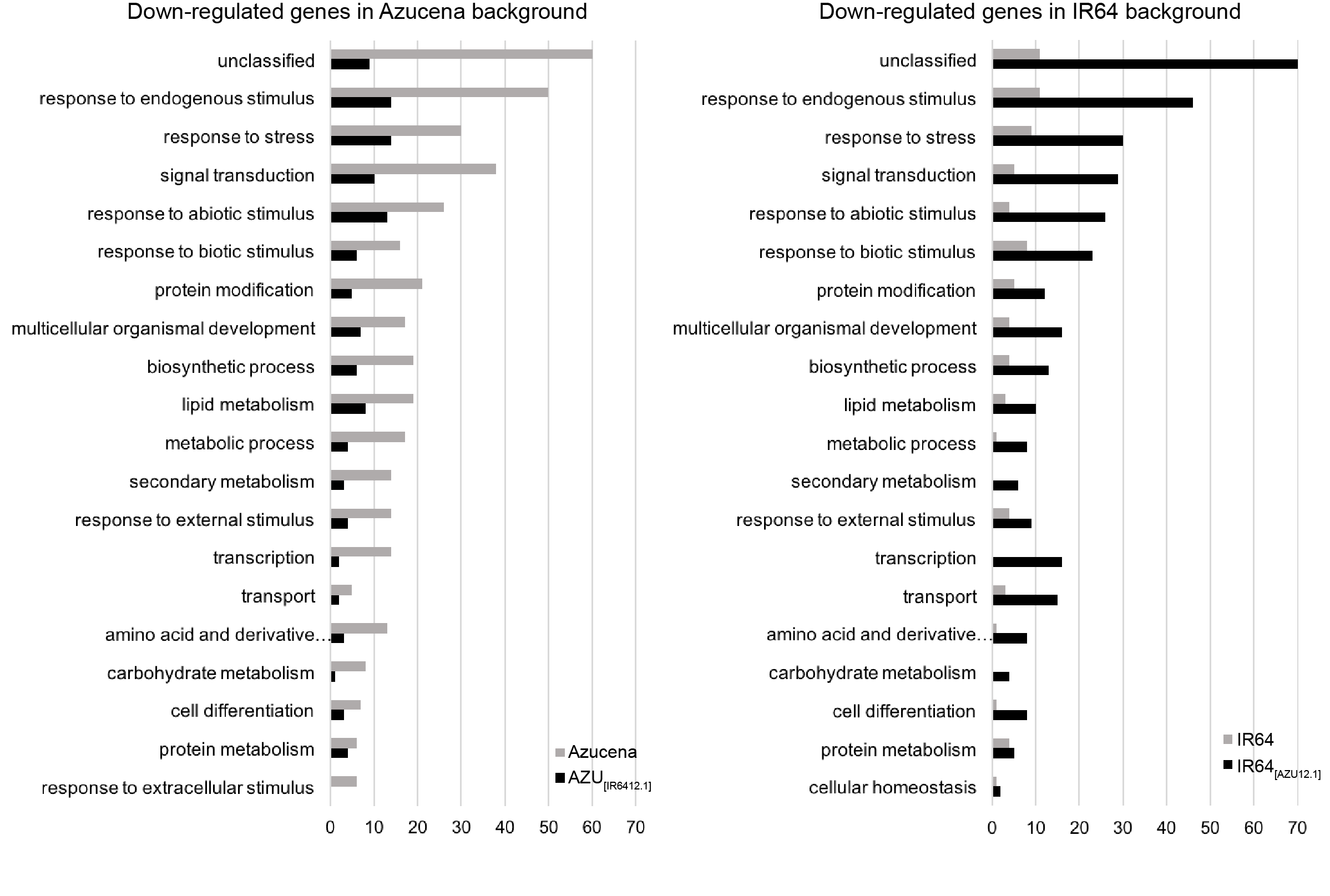


**Supplemental Figure S5:** **GO analysis of down-regulated genes in response to Al in Azucena (left) and IR64 (right) backgrounds.** The histograms display the number of differentially-regulated genes in each GO Process category.


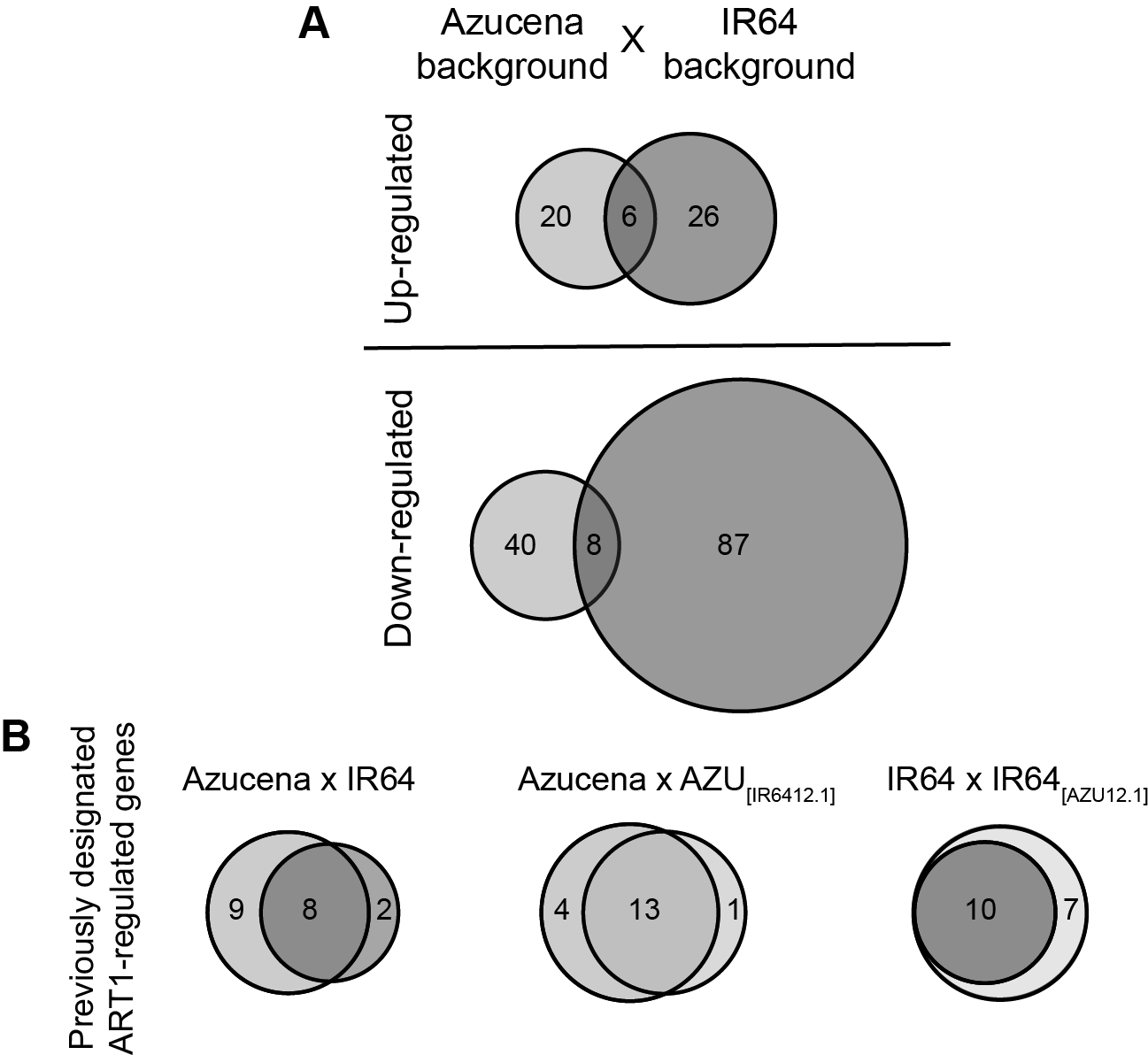


**Supplemental Figure S6:** The presence of the *ART1* allele from Azucena may not affect the same set of genes in *indica* and *japonica* backgrounds. **(A)** Venn diagram showing the overlap between the genes displayed at the top of the heat maps in Figure 6, in other words, the genes that respond more strongly to the *ART1* allele from Azucena. **(B)** Venn diagrams showing the presence of genes previously designated as ART1-regulated by Yamaji et al (2009) in our study.

**Supplemental Figure S7: *OsFRDL4* expression is affected by the different ART1 alleles i
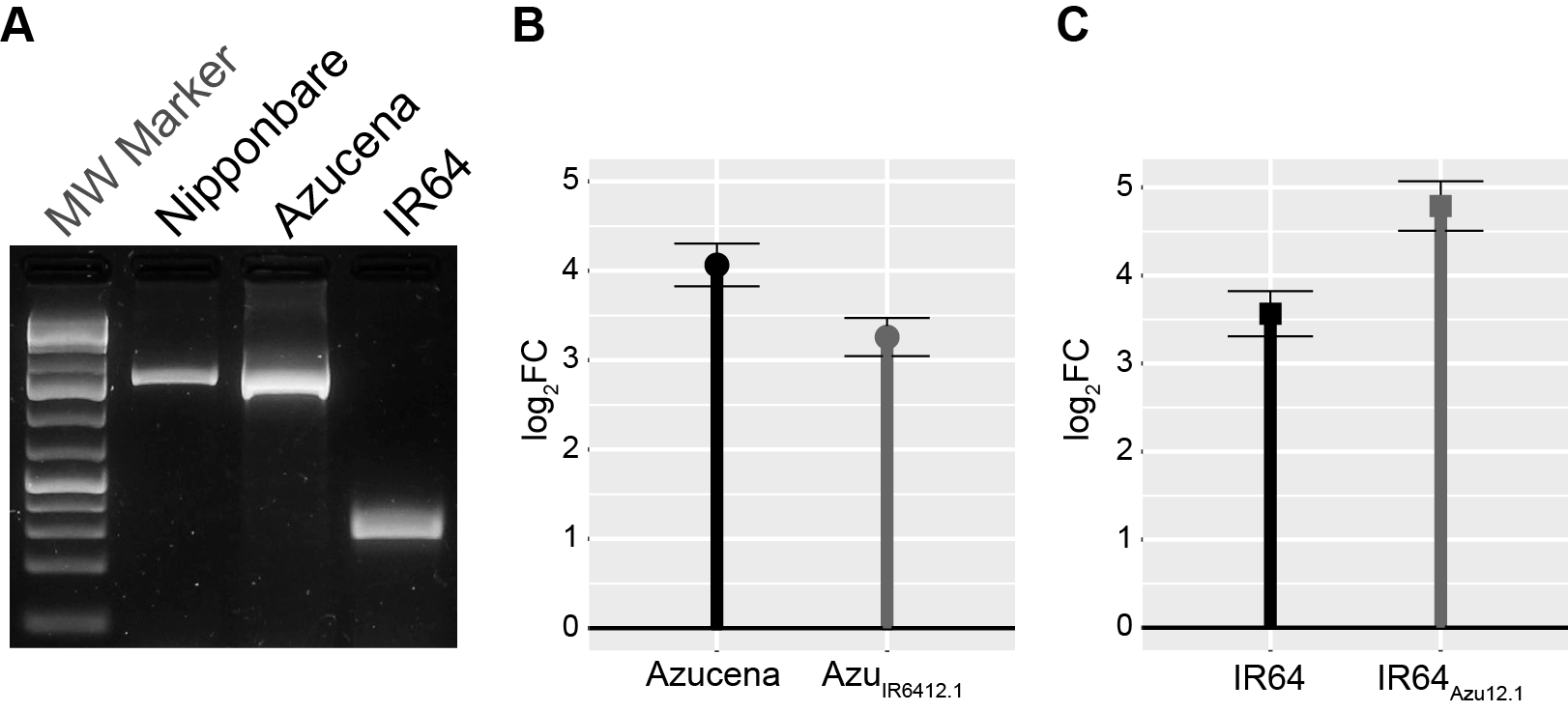
n Azucena and IR64 genetic backgrounds. (A)** PCR amplification of the *OsFRDL4* promoter region with primers flanking the 1.2-Kb insertion. The insertion is present in Nipponbare and Azucena, and absent in IR64. RNAseq-based expression pattern for *OsFRDL4* in response to Al (log_2_Fold-Change) in **(B)** Azucena and **(C)** IR64 genetic backgrounds.

**Supplemental Figure S8: Occurrence of ART1 predicted binding sites in putative regulatory regions of rice genes.** Distribution of the sequence motifs **(A)** “GGNVS” and **(B)** “GGYMS” in the putative regulatory regions (2 Kb upstream of start codon) of all rice gene models. There are 55,986 predicted loci in the rice genome (Nipponbare reference genome, MSUv.7), unmapped loci were excluded from the analysis (total used: 55,554). Mapping of motifs was performed using MotifMapper (Berendzen *et al.*, 2012). Only motifs in the forward orientation were counted.

Number of cis elements

Number of cis elements

**Methods S1. Developing of *Alt12*.*1* reciprocal near isogenic lines (NILs)**

IR64 and Azucena were used as recurrent parents for the development of the reciprocal NILs. Two lines from the IR64 x Azucena RIL mapping-population, RIL-48*,* and RIL-241, were selected as donor parents. The genetic composition of each donor RIL is 62.83% and 35.73% of IR64, 37.17% and 64.27% of Azucena respectively. RIL-48 was used as Azucena alleles donor for the *Alt12.1* locus for crossing into the IR64 recipient background, and RIL-241 was used as donor of *Alt12.1* IR64 alleles for crossing into the Azucena background.

A marker-assisted backcrossing (MABC) strategy using the indels ‘*indel_3.18*’, ‘*indel_ART1*’, and ‘*indel_3.62*’ (Supplemental Table S2) was conducted to develop each reciprocal NIL. For genotyping, total DNA was extracted from fresh leaf tissue using the Extract-N-Amp™ Plant Kit (Sigma-Aldrich, <http://www.sigmaaldrich.com/>). InDel primers were identified and designed according to Imai et al. (2013). Amplification and visualization of InDel markers were performed as described by Arbelaez et al. (2015). Three rounds of MABC and one cycle of selfing were performed to obtain homozygous NILs for *Alt12.1* in the IR64 (IR64_[AZU12.1]_) and Azucena (AZU_[IR6412.1]_) genetic background. Plants selected based on positive or forward selection using the set of three indels were then genotyped for negative or background selection, using a custom designed 6K-Infinium-array that assayed 2,387 SNP markers distributed across the rice genome. Plants that contained positive donor alleles in the target regions and as few negative donor alleles as possible in the remaining genetic background were self-pollinated and/or advanced to the next cycle of back-crossing.

To validate the *Alt12.1* QTL effects, phenotypic evaluation for Al resistance was performed in hydroponic growth media using the screening platform and root-imagining system described by Famoso et al. (2010) and Clark et al. (2013). Around 120 seeds of IR64, IR64_[AZU12.1]_, AZU_[IR6412.1]_, and Azucena were germinated under the same light and temperature conditions as described by Famoso et al. (2010). For each genotype, 20 uniform seedlings based on their primary root length were transplanted into modified Magnavaca hydroponic solution without stress (0 μM of Al^3+^ activity) and with Al stress (160 μM of Al^3+^ activity) and grown for 5 days. Photographs were taken to quantify root growth on each individual plant (as per Clark et al. 2013). Root images for Al resistance evaluation were taken and processed as described by Famoso et al. (2010) and Clark et al. (2013). The relative root growth (RRG) indices were estimated as described by Famoso et al. (2010).

Pairwise comparisons among RRG phenotypic values on each reciprocal NIL and their parents were performed using one-way analysis of variance (ANOVA) in R ([www.r-project.org/](http://www.r-project.org/)). Contrasts among NILs with each parent were performed using a Tukey’s HSD multiple comparison test with a significant threshold set of 5% (*P* < 0.05). Levels not connected by the same letter are significantly different (Figure 1D).

**Methods S2.** **Fine mapping of *Alt12.1*.**

**Development of *Alt12.1* fine-mapping population**

The elite rice variety IR64 and the recombinant inbred line (RIL) RIL-48*,* from the IR64 (*O. sativa* ssp. *indica*) x Azucena (*O. sativa* ssp. *japonica*) RIL mapping-population (Ahmadi et al. 2005) were used as starting parental material to develop two fine mapping populations. RIL-48 contains Azucena alleles across the *Alt12.1* region on chromosome 12 and its genetic makeup consists of 62.83% IR64 and 37.17% Azucena. RIL-48 was backcrossed with IR64 and 20 F_1_ plants were selfed to generate F_3_ and F_4_ populations for fine mapping of the *Alt12.1* locus. Seventeen molecular markers were used for fine mapping (Supplemental Table S2, Supplemental Figure S1).

Plants were genotyped using InDel and SNP markers based on competitive allele-specific PCR KASP™ chemistry assays (LGC, [www.lgcgroup.com/](http://www.lgcgroup.com/)). DNA was extracted from leaf tissue using the Extract-N-Amp™ Plant Kit (Sigma-Aldrich, [www.sigmaaldrich.com/](http://www.sigmaaldrich.com/)). InDel primers were designed, amplified and visualized as described in Methods S1. KASP™ SNP primers were identified and designed according to Imai et al. (2013). PCR conditions and graphical viewing of genotyped KASP™ markers were carried out as described by Imai et al. (2013).

A substitution mapping approach was implemented as described by Paterson et al. (1990) and Li et al. (2004) to localize and narrow down the candidate region associated with Al resistance. Recombinant lines were identified and grouped into ‘genotypic-groups’. Each genotypic group consisted of lines that shared the same informative recombination break point or ‘informative marker’ and overlap with a small fragment to the left or to the right of the informative marker (Suppl. Fig. S1). To associate significant phenotypic differences with graphical genotypes, pairwise evaluations for Al resistance were performed by examining graphical genotypes within each genotypic group with their Al resistance phenotypic values using progenies derived from each line (F_3:4_ and F_4:5_ generation) (Suppl. Fig. S1).

**Fine mapping of the *Alt12.1* locus**

A total of 4,224 F_3_ plants derived from a cross between RIL-48 and IR64 were genotyped with 11 molecular markers across a 2,400 Kb (12 cM) region encompassing the *Alt12.1* locus (Suppl. Fig. S1). Of these, 481 recombinant plants were identified and grouped into five ‘genotypic groups’. Pairwise phenotypic evaluations of their progenies were carried out between specific ‘genotypic groups’. As controls, non-recombinant F_3_ lines across *Alt12.1* were included in the analysis (‘CONTROL 1’).

Phenotypic evaluation of Al resistance, statistical analysis and pairwise comparisons among Azucena, IR64, and *Alt12.1* parental and recombinant lines (‘genotypic groups’) in the fine-mapping analysis, were performed as described in Methods S1. Significant differences in Al resistance based on RRG were observed when the progeny of homozygous lines carrying Azucena alleles (AA), heterozygous lines (het), and homozygous lines carrying IR64 alleles (II) were compared (ANOVA, *P* < 0.05). AA lines were significantly more resistant (RRG mean = 0.64) than II (RRG mean = 0.49) (Suppl. Fig. S1). The mean phenotype of the *het* lines (RRG mean = 0.56) was not significantly different than the mid-parent value estimated between the AA and II lines (RRG mean = 0.565), indicating that the *Alt12.1* QTL had an additive effect.

The first genotypic group, ‘GROUP 1’, was used to test if the region associated with Al resistance in the *Alt12.1* QTL was to the left or right of the marker ‘*K_3.28’*. The RRG of 120 plants derived from lines genotyped AA and het to the left and right of ‘*K_3.28’*, respectively (RRG mean = 0.52), now referred as AA-K_3.28-het plants, was compared with the RRG of 133 plants derived from lines genotyped II-K_3.28-het (RRG mean = 0.55). In ‘GROUP 2’, 176 plants derived from lines genotyped het-K_3.28-AA (RRG mean = 0.65) were compared with 80 plants genotyped het-K_3.28-II (RRG mean = 0.37). Pairwise tests with a significant level of *P* < 0.05 in ‘GROUP 1’ indicated no significant differences between AA-K_3.28-het and II-K_3.28-het lines. Comparison in ‘GROUP 2’ between het-K_3.28-AA and het-K_3.28-II showed significant differences for Al resistance. In conclusion, the locus associated with Al resistance is to the right of ‘*K_3.28*’.

‘GROUP 3’ was used to test if the *Alt12.1* QTL region associated with Al resistance was to the left or right of marker ‘*indel_3.62*’. The RRG of 346 plants derived from lines genotyped het-indel_3.62-II (RRG mean = 0.56), were compared with 410 plants derived from the contrasting genotype II-indel_3.62-het (RRG mean = 0.46). Pair wise tests between the lines in ‘GROUP 3’ showed significant differences. When they were compared with control lines het and II from ‘CONTROL 1’ it was concluded that the locus associated with Al resistance was to the left of ‘indel_3.62’. From these analyses, it was concluded that a region of 340 Kb between markers ‘*K_3.28*’ and ‘*indel_3.62*’ is associated with Al resistance (Suppl. Fig. S1).

A total of 3,552 informative F_4_ plants were used for the second round of fine-mapping. These individuals were genotyped with 6 molecular markers targeting a region of 340 Kb (1.7 cM). Based on this analysis, we identified 55 recombinants and grouped them into five ‘genotypic groups’. To test if the region associated with Al resistance was to the left or right of marker ‘*K_3.57*’, 152 plants derived from lines with the genotype II-K_3.57-het and phenotyped for Al resistance (RRG mean = 0.56), were compared with 59 plants derived from lines with the genotype AA-K_3.57-II (RRG mean = 0.48) from ‘GROUP 5’, 148 het, and 113 II control plants from ‘CONTROL 2’ group. Pairwise tests (*P* < 0.05) showed significant differences between II-K_3.57-het compared to AA-K_3.57-II and control lines II. It was concluded that the locus associated with Al resistance was to the right of marker ‘*K_3.57*’. Based on these results, a target region of 44.74 Kb between markers ‘*K_3.57*’ and ‘*indel_3.62*’ was determined to be associated with Al resistance. The target region is located between 3,578,414 - 3,623,153 bp on chromosome 12.

**Table S1:** Genomic composition and Al resistance phenotype of the reciprocal NILs.

| **Line name** | **Number of donor segments** | **% Recurrent genome** | **% Donor genome** | **Chr. with segments** | **Target segment position MSU.7 (Mb)** | **Target segment size (Mb)** | **Non-target segment size (Mb)** | **N** | **RRG mean or TRG mean** | **Lower 95%** | **Upper 95%** | ***TK-HSD**  **(*P < 0.05*)** |
| --- | --- | --- | --- | --- | --- | --- | --- | --- | --- | --- | --- | --- |
| **Azucena** | n.a. | 100.00 | 0.00 | n.a. | n.a. | n.a. | n.a. | 18 | 0.89 | 0.84 | 0.94 | **A** |
| **AZU_[IR6412.1]_** | 1 | 98.95 | 1.05 | 12 | 2.20 - 6.13 | 3.92 | n.a. | 18 | 0.62 | 0.56 | 0.66 | **B** |
| **IR64_[AZU12.1]_** | 1 | 99.37 | 0.63 | 12 | 1.35 - 3.72 | 2.36 | n.a. | 18 | 0.66 | 0.61 | 0.71 | **B** |
| **IR64** | n.a. | 100.00 | 0.00 | n.a. | n.a. | n.a. | n.a. | 18 | 0.56 | 0.50 | 0.61 | **C** |

*TK-HSD (*p < 0.05*): Tukey-Kramer HSD test, levels not connected by the same letter are significantly different (*P < 0.05*).

**Table S2:** Molecular markers used during fine-mapping analysis.

| **Marker ID** | **Chr** | **Position MSUv7 (bp)** | **Forward** | **Reverse** |
| --- | --- | --- | --- | --- |
| ***K_2.93*** | 12 | 2940317 | *GAAGGTGACCAAGTTCATAGACA | ATAAGCTGAAAACCTGGAAACAGTGG |
|  |  |  | **GAAGGTCGGAGTCAACGGATTCAGA |  |
| ***indel_3.18*** | 12 | 3186609 | TGCCAAAGCGCAGAGAGAGA | TAGTGCCGATCGATGTAACG |
| ***K_3.28*** | 12 | 3287769 | *GAAGGTGACCAAGTTCATGCTATTAT | CTCCTTCGTCTTGCTCATTGGCATT |
|  |  |  | **GAAGGTCGGAGTCAACGGATTATTCA |  |
| ***K_3.4*** | 12 | 3480237 | *GAAGGTGACCAAGTTCATGCTGTGAA | GCCTGCCGTTCACGCTGTACAA |
|  |  |  | **GAAGGTCGGAGTCAACGGATTGAATG |  |
| ***K_3.57*** | 12 | 3578363 | *GAAGGTGACCAAGTTCATGCTACACTCCTGATTTTATAGTGTAATGTTTG | GCAAAAGACATCGGGCTCATGTCAA |
|  |  |  | **GAAGGTCGGAGTCAACGGATTCACACTCCTGATTTTATAGTGTAATGTTTA |  |
| ***indel_ART1*** | 12 | 3581918 | CGACGAGCTCTTCAAGGTATG | GCAGCACCTCGTACTTCTCC |
| ***K_3.58*** | 12 | 3586738 | *GAAGGTGACCAAGTTCATGCTATTCT | CTCCGTGAGTTGGCATCATATGTGAA |
|  |  |  | **GAAGGTCGGAGTCAACGGATTCTGCA |  |
| ***indel_3.62*** | 12 | 3623299 | GCACACACCATTAAGTCAAACAA | TCCACCAAAGATTAAGGCTGA |
| ***K_3.8*** | 12 | 3865251 | *GAAGGTGACCAAGTTCATGCTATCGA | ACTAACCTGAACATGTCATGCAGAGA |
|  |  |  | **GAAGGTCGGAGTCAACGGATTGATC |  |
| ***indel_4.14*** | 12 | 4141713 | CGATCTAGTCGACAACTGCAA | TGGAACAGTACGTTGTGATCTTC |
| ***indel_4.64*** | 12 | 4649818 | CCGTTTCTGCTACACTTTCTCTTT | ACCGAACACACAATTTCAGATG |
| ***indel_4.75*** | 12 | 4760008 | ATCTTGAGAAGTTGTCCCTTGATT | CCTATCAAATGTAACACAAGCACA |
| ***indel_4.98*** | 12 | 4981850 | ATCTGTGAAGGAGGGAGGTGT | CTCTATTTCTTGTTGATTACAGGATCA |

* 1^st^ allele-specific forward primer

** 2^nd^ allele-specific forward primer

**Table S3:** Statistics for the RNA-seq read alignments.

| **Rice line** | **Treatment** | **Rep** | **Raw reads** | **Clean reads** | **Uniquely mapped reads** | **% Uniquely mapped reads MSU7.0** |
| --- | --- | --- | --- | --- | --- | --- |
| **Azucena** | Al | 1 | 17948752 | 15155305 | 11124922 | 73.41 |
| **Azucena** | Al | 2 | 14505176 | 12330353 | 9178901 | 74.44 |
| **Azucena** | Al | 3 | 16449078 | 13807061 | 10980422 | 79.53 |
| **Azucena** | Al | 4 | 16353541 | 14017553 | 10796821 | 77.02 |
| **Azucena** | CONTROL | 1 | 17522810 | 14772600 | 10965244 | 74.23 |
| **Azucena** | CONTROL | 2 | 18406152 | 15807826 | 11486446 | 72.66 |
| **Azucena** | CONTROL | 3 | 19316475 | 16295079 | 11665107 | 71.59 |
| **Azucena** | CONTROL | 4 | 18681017 | 15966724 | 12676932 | 79.4 |
| **AZU_[IR6412.1]_** | Al | 1 | 21093395 | 17669638 | 12378978 | 70.06 |
| **AZU_[IR6412.1]_** | Al | 2 | 18312476 | 15681495 | 11474016 | 73.17 |
| **AZU_[IR6412.1]_** | Al | 3 | 20487685 | 17093152 | 13682821 | 80.05 |
| **AZU_[IR6412.1]_** | Al | 4 | 18137649 | 15403258 | 12201747 | 79.22 |
| **AZU_[IR6412.1]_** | CONTROL | 1 | 19598070 | 16529872 | 11979654 | 72.47 |
| **AZU_[IR6412.1]_** | CONTROL | 2 | 18916056 | 16090084 | 11563131 | 71.86 |
| **AZU_[IR6412.1]_** | CONTROL | 3 | 15897060 | 13136827 | 10385578 | 79.06 |
| **AZU_[IR6412.1]_** | CONTROL | 4 | 17363828 | 14856336 | 11700792 | 78.76 |
| **IR64** | Al | 1 | 24899721 | 20795049 | 18070433 | 86.9 |
| **IR64** | Al | 2 | 17555466 | 14943875 | 10540272 | 70.53 |
| **IR64** | Al | 3 | 19033733 | 15752119 | 11433341 | 72.58 |
| **IR64** | Al | 4 | 20302634 | 17471828 | 12870027 | 73.66 |
| **IR64** | CONTROL | 1 | 13755843 | 11580249 | 7951241 | 68.66 |
| **IR64** | CONTROL | 2 | 16697075 | 13838746 | 7962725 | 57.54 |
| **IR64** | CONTROL | 3 | 15829135 | 12990740 | 10033086 | 77.23 |
| **IR64** | CONTROL | 4 | 20623409 | 17250638 | 12061320 | 69.92 |
| **IR64_[AZU12.1]_** | Al | 1 | 18763298 | 15937902 | 11352778 | 71.23 |
| **IR64_[AZU12.1]_** | Al | 2 | 17996234 | 15245383 | 9919338 | 65.06 |
| **IR64_[AZU12.1]_** | Al | 3 | 19073259 | 15859854 | 11447661 | 72.18 |
| **IR64_[AZU12.1]_** | Al | 4 | 18600545 | 15850418 | 11893442 | 75.04 |
| **IR64_[AZU12.1]_** | CONTROL | 1 | 17207685 | 14453667 | 9714534 | 67.21 |
| **IR64_[AZU12.1]_** | CONTROL | 2 | 16158737 | 13781495 | 9463233 | 68.67 |
| **IR64_[AZU12.1]_** | CONTROL | 3 | 17053995 | 14273789 | 9544607 | 66.87 |
| **IR64_[AZU12.1]_** | CONTROL | 4 | 18122425 | 15521949 | 11695533 | 75.35 |
| **Total** |  | **32** | **580662414** | **490160864** | **360195083** |  |
| **Average** |  |  | **18145700.44** | **15317527** | **11256096.34** | **73.29875** |

**Table S4:** SNPs genotyped for RNA-seq sample ID verification across 14 loci in Azucena, IR64, and the NILs AZU_[IR6412.1]_ and IR64_[AZU12.1]_.

| **Gene model** | **Chr.** | **Exon** | **IR64 allele** | **Azucena allele** | **IR64_[AZU12.1]_ allele** | **AZU_[IR6412.1]_ allele** |
| --- | --- | --- | --- | --- | --- | --- |
| LOC_Os01g69010.1 | 1 | Exon.2 | **T** | **G** | **T** | **G** |
| LOC_Os02g51930.1 | 2 | Exon.2 | **G** | **A** | **G** | **A** |
| LOC_Os03g54790.1 | 3 | Exon.17 | **T** | **C** | **T** | **C** |
| LOC_Os04g49410.1 | 4 | Exon.3 | **G** | **A** | **G** | **A** |
| LOC_Os05g02750.1 | 5 | Exon.1 | **A** | **G** | **A** | **G** |
| LOC_Os06g48060.1 | 6 | Exon.4 | **T** | **C** | **T** | **C** |
| LOC_Os07g37730.1 | 7 | Exon.3 | **A** | **G** | **A** | **G** |
| LOC_Os08g39450.1 | 8 | Exon.1 | **C** | **A** | **C** | **A** |
| LOC_Os09g26170.1 | 9 | Exon.3 | **T** | **C** | **T** | **C** |
| LOC_Os10g13940.1 | 10 | Exon.4 | **T** | **C** | **T** | **C** |
| LOC_Os11g06720.1 | 11 | Exon.1 | **C** | **T** | **C** | **T** |
| LOC_Os12g07280.1 | 12 | Exon.2 | **A** | **G** | **G** | **A** |
| LOC_Os12g07310.1 | 12 | Exon.2 | **C** | **T** | **T** | **C** |
| LOC_Os12g07670.1 | 12 | Exon.6 | **A** | **G** | **G** | **A** |
